# Fellow-Workers and the Roll of Honour

**Published:** 1915-03-06

**Authors:** 


### ROUND THE WORLD.

[The Editor invites Early Information and Contributions Marked "Fellow-Workers."]

### Fellow-Workers and the Roll of Honour.

The ophthalmic department of the King George Hospital is under the direction of four well-known London ophthalmic surgeons—namely, Sir G. Anderson Critchett, Sir W. J. Collins, Mr. Ernest Clarke, and Mr. William Lang.

Mr. Ernest Clarke is consulting surgeon to the Central London Ophthalmic Hospital and to the Miller Hospital at Greenwich. A Cambridge and "Bart.'s" man, he took the M.D., B.S. Lond., and F.R.C.S. Eng. Formerly a vice-president of the Ophthalmological Society and president of the West Kent Medico-Chirurgical Society, he has written largely on ophthalmic subjects, including a work for students on "The Refraction of the Eye," which has run through several editions. In early years he won exhibitions in science both at St. Bartholomew's Hospital and at Cambridge, where he was for a time assistant demonstrator in anatomy.

Sir William Job Collins, K.C.V.O., F.R.C.S., Eng., M.D., M.S. Lond., is consulting surgeon to the Western Ophthalmic Hospital and to the London Temperance Hospital, and has done public work of considerable importance. He distinguished himself as a student of St. Bartholomew's, winning a university scholarship and gold medal in obstetric medicine with first-class honours in forensic medicine at the M.B. Lond., honours at the B.S. Lond., and honours in physiology at the B.Sc. Lond. He subsequently held numerous important public appointments, including those of chairman of the London County Council, vice-chancellor of the University of London, and chairman of the London Education Committee. Sir William Collins was knighted in 1902, and made a K.C.V.O. last year. He was M.P. for West St. Pancras from 1906 to 1910.

Sir George Anderson Critchett is the distinguished son of a distinguished father who himself enjoyed a great reputation as an ophthalmologist. Proceeding to Middlesex Hospital from Cambridge as a student of medicine, he qualified M.R.C.S. Eng., and subsequently became a Fellow of the Royal College of Surgeons of Edinburgh. Specialising in ophthalmic surgery, he was appointed a member of the staff of St. Mary's Hospital, to which institution he is now consulting ophthalmic surgeon. Surgeon-oculist to his Majesty the King, and ophthalmic surgeon to the King Edward VII. Hospital, Sir Anderson Critchett is a past-president of the Ophthalmological Society of the United Kingdom, and was at one time ophthalmic surgeon to the Royal Free Hospital. He was knighted in 1901 and created a baronet in 1908.

Mr. William Lang, F.R.C.S. Eng., is a London Hospital man, who, specialising in ophthalmology, became attached to the Middlesex and Royal London Ophthalmic Hospitals, to both of which institutions he became consulting ophthalmic surgeon after many years' work on their active staffs. In his early years Mr. Lang, who enjoys a world-wide reputation as an ophthalmologist, was house surgeon and house physician at the London Hospital, and subsequently demonstrator in pathology and anatomy in the medical school there.

Dr. Florence Ada Stoney, who is in charge of the Anglo-French Hospital of the Imperial Service League, near Cherbourg, is an expert in electro-therapeutics and radiography. A student of the London School of Medicine for Women, she became an M.D. B.S. Lond., and after serving various junior appointments, including that of house surgeon to the Victoria Hospital at Hull, was appointed director of the electrical department at the New Hospital for Women. Dr. Stoney has under her six other lady doctors and fifteen nurses.

Three members of the staff of the Royal Victoria Hospital at Belfast have joined the hospital which the Red Cross Society is sending abroad in the spring, and have for this purpose obtained the necessary leave of absence. They are Dr. T. Houston, Dr. J. E. MacIlwaine, and Mr. P. T. Crymble.

Dr. Thomas Houston is physician in charge of the department for hæmatology and vaccine therapy at the Royal Victoria Hospital, pathologist to the Ulster Hospital, and visiting physician to the Forster Green Hospital. A student of Queen's College, Belfast, he qualified M.B., B.Ch. in 1895, subsequently taking the M.D. of the Royal University of Ireland, where he is University student in pathology. Dr. Houston, who was formerly a research scholar of the British Medical Association, is joint lecturer in medical jurisprudence at Queen's College, Belfast, and has held various junior appointments at the Royal Victoria Hospital, including those of assistant gynæcologist, assistant pathologist, and medical registrar.

Dr. John Elder MacIlwaine is assistant physician to the Royal Victoria Hospital, Belfast, where he was at one time house surgeon and house physician, and also visiting physician to the Forster Green Hospital there. He studied medicine at Belfast, Dublin, Vienna, Berlin, and Paris, and after taking first-class honours and exhibitions at his qualifying degree, won the gold medal at the M.D. examination of the Royal University of Ireland in 1904; three years later he became a D.P.H. Cambridge. Dr. MacIlwaine has had previous experience of military work, as he was medical officer to the Irish Hospital during the South African War.

Mr. Percival Templeton Crymble, M.B., B.Ch. R.U.I., F.R.C.S. Eng., in addition to his appointment as surgical registrar to the Royal Victoria Hospital at Belfast, is out-patient surgeon to the Hospital for Sick Children there and lecturer on applied anatomy at Queen's College A student of the London University as well as of Belfast, Mr. Crymble won the Mackay Wilson travelling scholarship in 1909. His past appointments in Belfast include those of assistant pathologist and honorary anæsthetist to the Royal Victoria Hospital, and senior demonstrator in anatomy at Queen's College.

Captain Sidney Clement Bowle, R.A.M.C., M.R.C.S., L.R.C.P., mentioned in dispatches, is a "Guy's" man, who took both medical and dental diplomas, and in his early years acted both as dental house surgeon and ophthalmic house surgeon there. Subsequently entering the R.A.M.C., his name has now been brought prominently before the public for his fine work on the field of battle.

